# Impact of dehulling, germination and fermentation on the bioactive and functional properties of grey pea flour

**DOI:** 10.3389/fnut.2024.1478399

**Published:** 2024-10-09

**Authors:** Armaghan Amanipour, Yasaman Samaei, Olof Böök, Yvonne Granfeldt, Claudia E. Lazarte

**Affiliations:** ^1^Division of Food and Pharma, Department of Process and Life Science Engineering, Faculty of Engineering, Lund University, Lund, Sweden; ^2^Aventure AB, Lund, Sweden

**Keywords:** antioxidant capacity, fermentation, functional properties, germination, grey pea, polyphenols

## Abstract

**Introduction:**

Grey pea is a largely overlooked legume in the Nordic countries, and its potential uses in various food products remain unexplored. It is a nutrient-rich crop with low environmental impact, making it an attractive option for sustainable and nutritious plant-based alternatives.

**Objectives:**

To investigate the impact of dehulling, germination, and fermentation on the bioactive (polyphenol content and antioxidant capacity) and functional characteristics (water absorption index, water solubility index, water and oil binding capacity, emulsifying properties and gelation concentration) of grey pea flour. Additionally, protein content and pasting properties (temperature, peak viscosity, trough viscosity, breakdown, final viscosity, and setback) were measured.

**Methods:**

Dehulling was performed using a runner disk sheller. Germination was carried out for 24 and 48 h at ambient temperature, and fermentation was conducted for 8 h at 43°C using a starter culture.

**Results:**

The results indicate that dehulling did not significantly affect functional properties and gelling capacity (*p* = 0.297 for oil absorption capacity, *p* = 0.5 for emulsion activity, and *p* = 0.607 for emulsion stability), but it resulted in a notable decrease in total polyphenol content (TPC) and antioxidant capacity (TAC). Conversely, 48 h of germination increased TAC measured by two methods: FRAP (19%) and DPPH (30%). This process increased through viscosity by 1.2-fold, while it did not significantly affect the water absorption index (WAI), water solubility index (WSI), or the emulsifying properties of grey pea flour. Fermentation significantly improved TPC (*p* < 0.001 for whole grey peas and *p* = 0.004 for dehulled grey peas), with a TPC increase of up to 67% in fermented dehulled pea flour. TAC measured by both methods, showed significant increases, ranging from 35 to 104%. However, fermentation reduced emulsifying and pasting properties, as indicated by the peak, through and final viscosity, which may be desirable only for certain food products. Further, germination and fermentation showed significant increases in protein content, by 4 and 8%, respectively.

**Conclusion:**

Fermented grey pea flour exhibited enhanced bioactive characteristics, while 48-h germination positively impacted pasting properties. Overall, these processes led to changes in both the bioactive and functional properties of grey pea flour, creating opportunities for the use of these flours in a wide array of food products.

## 1 Introduction

Grey pea, a subgroup of the peas (*Pisum sativum L*.) and pulses family, was once a staple food in the Nordic countries used in various dishes from soup to bread. However, its popularity declined from the 19th century until today (1). Nowadays, grey pea is regaining attention due to its status as a nutrient-rich crop, it has high protein, fiber and mineral content. Besides its nutritional properties, grey peas can be sustainably cultivated, improving soil fertility through nitrogen-fixing properties and intercropping with cereals like oats, thus increasing farm biodiversity. Tidåker et al. (2) showed that the environmental impact of cultivation of grey pea was lower than that of beans. Thus, the growing awareness and interest in improving overall health and minimizing the environmental impact of dietary choices has led to more attention toward the consumption of pulses. Recent trends in Sweden regarding the healthy eating and consumption of sustainable and locally sourced foods as well as increasing interest in a more plant-based diet have promoted the consumption of domestically grown pulses such as grey peas (3). It is believed that its cultivation can be expanded and it has the potential to reduce dependency on imported soybean in the future (4). However, up to date the consumption of grey peas is still limited, within one of the limitations is the lack of information on the functional properties of these peas along with information on how processing alters the nutritional and bioactive compounds in peas.

Several processing methods, including dehulling, cooking, roasting, germination, and fermentation, are employed to mitigate antinutrient levels, enhance palatability, and improve sensory acceptance of pulses and pulse flours. These treatments not only increase the bioavailability of nutrients in pulses but have the potential to enhance their functional properties (3, 5). Dehulling is a process of loosening and removal of the fibrous seed coat (6). This process improves the appearance, cooking quality, and palatability of the pulses as well as enhancing their digestibility (7). Additionally, dehulling produces higher quality flour without visual specks. Germination is a traditional and cost-effective process that enhances the nutritional and functional properties of pulses. During germination structural elements are altered and novel bioactive compounds are synthesized which improves digestibility, stability, and nutritional profile of the grains (8). Moreover, sensory properties may improve during germination by reducing the beany flavor through the activation of endogenous enzymes and the conversion of starch into simpler sugars (9). Fermentation is an ancient food technology in which a population of microorganisms is utilized for biological conversion of complex substrates into simpler compounds (10). The fermentation process can be beneficial due to the elimination and decrease of antinutritional factors such as phytic acid, trypsin, and chymotrypsin inhibitors, thus improving the nutritional quality and protein digestibility of pulses (11).

Relatively extensive work has been undertaken investigating the effects of germination and fermentation on nutritional and antinutritional compounds in pulses. However, there is limited information on the effect of processing methods, such as germination and fermentation, on the production of bioactive compounds, i.e., polyphenols. Pulses are sources of phenolic compounds such as phenolic acid, flavonoids, isoflavones and tannins. The specific types and amounts of these phenolic compounds which may exist in free, esterified or bound forms differ depending on the type and genotypes of pulse (12). Fermentation has been shown to increase bioactive phenolic compounds in some pulses and legumes, resulting in greater antioxidant activities. Moreover, conjugated phenolic compounds can be converted into their free forms during fermentation enhancing their bioavailability and health benefits (13). Furthermore, changes in functional properties of grey pea flour during processing are not sufficiently reported. Functional properties play a crucial role in developing new food products and determining the behavior of food during manufacturing, processing, storage, and consumption (14). It was also reported that functional properties of food ingredients can enhance processing efficiency (15). For example, fermentation of sorghum flour improved functional properties such as emulsifying capacity and stability (16). Other authors noted that yeast fermentation of rice reduced the hot paste viscosity (17), highlighting the relevance of investigating changes in functional properties of flours due to processing, which can have a great impact on the use of these ingredients in the food industry.

Therefore, this study aimed at investigating the effect of dehulling, germination, and fermentation on protein content, total phenolic content and antioxidant capacity of grey pea flour. Moreover, physical, functional, and pasting properties of the untreated and treated flours were studied. This study will pave the way toward creating new food ingredients with improved health benefits and functional properties. The information on the raw and processed grey pea flour will shed light on the most suitable way to diversify the use of grey peas in the food industry.

## 2 Materials and methods

### 2.1 Materials

Dried grey peas (variety retrija) were purchased from Nordisk Råvara (Stockholm, Sweden). Starter culture Lyofast VSAB1 3UC/100 L (SACCO starter cultures, Kemikalia AB, Skurup, Sweden) was used for fermentation of grey peas. All reagents used were of analytical grade. Sodium hydroxide (VWR Chemicals, Leuven, Belgium) used for acidity. Folin–Ciocalteu reagent, sodium carbonate, gallic acid, TPTZ (1,3,5-tri(2-pyridyl)-2,4,6-triazine), sodium acetate, DPPH (2,2-diphenyl-1-picrylhydrazyl), and Trolox ((+/*−*)-6-Hydroxy-2,5,7,8-tetramethylchromane-2-carboxylic acid) were purchased from (Sigma-Aldrich, Stockholm, Sweden). Ferric chloride (FeCl_3_*·*6H_2_O) was purchased from (Fluka, Hanover, Germany).

### 2.2 Methods

#### 2.2.1 Processing of grey peas

**Figure 1:**
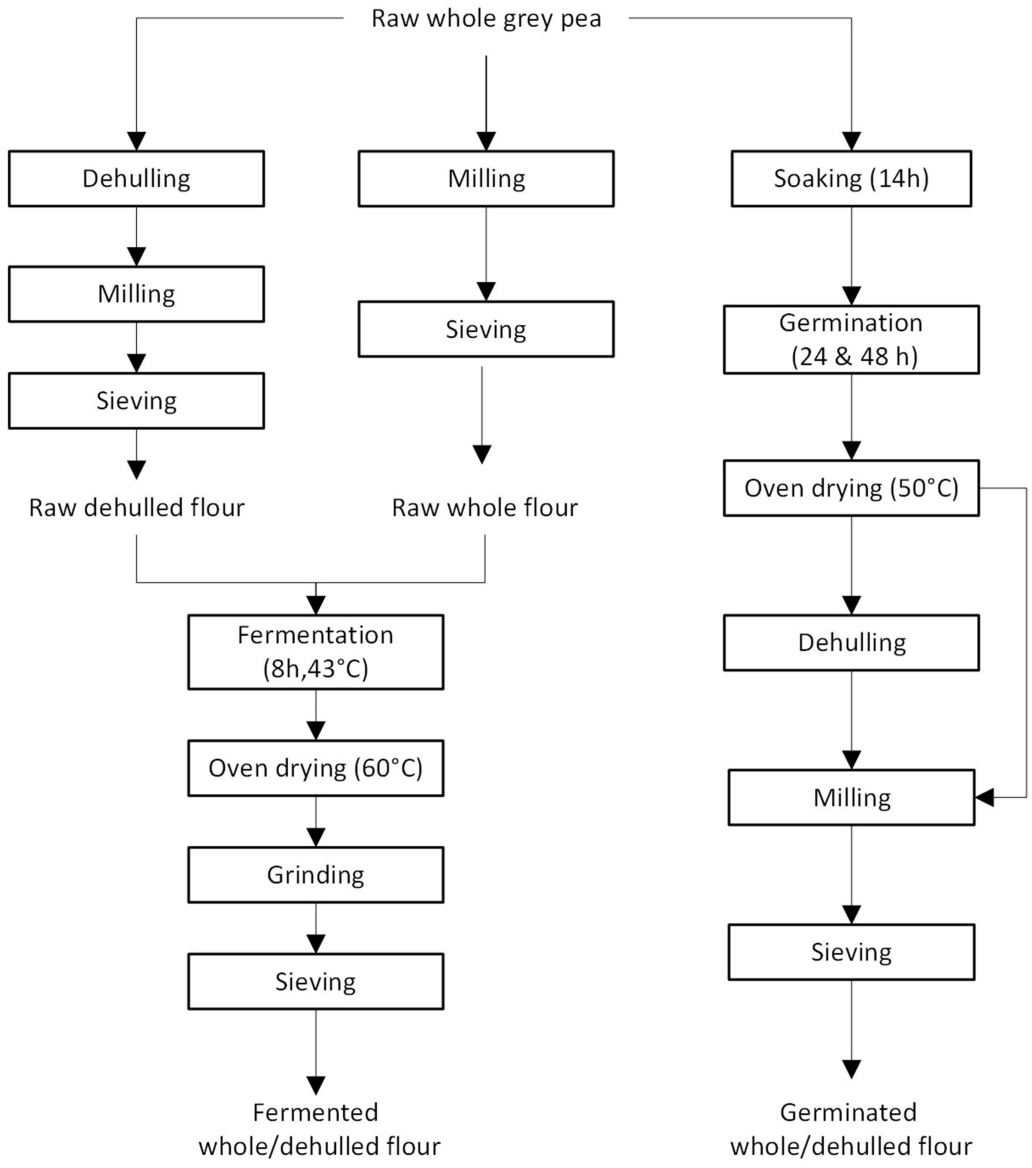
Flowchart of processes and conditions used in dehulling, germination and fermentation of grey peas.

Dehulling, germination, and fermentation were the methods used in processing grey peas. All the processes were conducted in duplicate. A flowchart of the processing steps is presented in Figure 1.

*Dehulling* of the unprocessed grey peas was performed using a runner disk sheller (Streckel and Schrader, Hamburg, Germany). Grey peas, either whole or dehulled were milled using a hammer mill (Laboratory mill 120, Perten Instruments AB, Stockholm, Sweden) and sifted through a 500 μm sieve. The flour was weighed into small portions, packed in the vacuum bags, and stored at 4°C for further analysis or processing.

*Germination* was conducted in whole grey peas following the method described by Ferawati et al. (3) with slight modification. In this study, the peas were first washed and then soaked in tap water (1:3 w/v) for 14 h at room temperature (~20°C), rather than under controlled temperature conditions in an incubator. Soaked grey peas were put between layers of wet tissue paper and germinated under ambient laboratory conditions for 24 and 48 h. The germinated seeds were divided into two batches, one to be used as whole and in the second batch, the peas were dehulled. The germinated peas (whole and dehulled) were dried in oven at 50°C (Termaks, TS4057, Bergen, Norway) and then milled to obtain the germinated pea flours.

*Fermentation* of grey pea flours (whole and dehulled) was conducted following the method standardized in our previous studies (18). Briefly a suspension of grey pea flour was prepared with distilled water (1:2 w/v) and inoculated with the starter culture Lyofast VSAB and incubated at 43°C for 8 h (Termaks, TS4057, Bergen, Norway). Lyofast VSAB is a commercial starter culture that consists of selected strains of *Streptococcus thermophilus* added with probiotic strains of *Lactobacillus acidophilus* and *Bifidobacterium animalis ssp. lactis* with an optimum growth temperature of 43°C. It is mainly used to produce fermented vegetable drinks and dairy alternatives like vegan yoghurt; however, it has shown some potential in its applications fermenting plant-based food. To control the development of fermentation, samples were taken every 2 h to measure the pH and total acidity. After fermentation, the fermented slurries were dried at 60°C in an oven (Termaks, TS4057, Bergen, Norway).

##### 2.2.1.1 pH and total titratable acidity (TTA)

The pH and total acidity were measured at every 2 h intervals of fermentation until the end of fermentation (8 h). pH was measured in duplicate by the method described by Nuobariene et al. (19). Briefly 10 g of sample was suspended in 90 mL distilled water and stirred for 4 min and then the pH was recorded with a pH meter (Mettler Toledo, Greifensee Switzerland). The total acidity was determined by titration with 0.1 N sodium hydroxide (20). Briefly, a 30 mL of aliquot of the homogenized sample prepared for pH measurement was taken and titrated with 0.1 N NaOH, using phenolphthalein as an indicator. The titration continued until a faint pink color persisted for 30 s.

#### 2.2.2 Protein analysis

The protein content of the grey pea flour samples, before and after processing, was measured using the dynamic flash combustion method (modified Dumas method) as described by Krotz et al. (74). 0.25 mg of dried sample was weighed and placed in the protein analyzer equipment (Thermo Scientific™ Flash™, EA 1112 series, MA, USA), the nitrogen to protein conversion factor of 6.25 was used. All the results are presented on a dry weight basis (dwb). The moisture content of grey pea flour was determined using the AOAC method (22). 5 g of flour samples were dried in the oven at 105°C (Termaks, TS4057, Bergen, Norway) until a constant weight was obtained. All analyses were conducted by duplicate.

#### 2.2.3 Total phenolic content (TPC)

Extraction of phenolic compounds was performed according to the method of Xu and Chang (75). Flour samples (0.5 g) were extracted with 5 mL aqueous acetone 50% (v/v) for 3 h at room temperature at 300 rpm (IKA, KS 130 basic, Staufen, Germany). The extraction was followed by keeping the samples for 12 h in the dark. The samples were then centrifuged at 3000 rpm for 10 min (Centrifuge 5804R, Eppendorf, Hamburg, Germany) and the supernatants were transferred to new tubes. 5 mL of extraction solvent was added to the residues and the extraction procedure was repeated. The two extracts were combined and stored at 4°C in the dark until analysis.

The TPC was determined in the extracts using the method of Xu and Chang (75). 50 μL of the extract, 3 mL of distilled water, 250 μL of Folin–Ciocalteu’s reagent, and 750 μL of 7% Na_2_CO_3_ were mixed and incubated at room temperature. After 8 min, 950 μL of distilled water was added to the mixture and left for 2 h at room temperature. The absorbance was measured at 765 nm using a spectrophotometer (PerkinElmer, LAMBDATM Bio+, MA, USA) with distilled water as blank. TPC results in dwb were calculated and expressed as gallic acid equivalents (mg of GAE/g sample).

#### 2.2.4 Total antioxidant capacity (TAC)

The extraction procedure was followed according to the method of Sulaiman et al. (24). Briefly 2 g of flour samples were extracted with 25 mL of 70% (v/v) acetone for 24 h in room temperature using a shaker set at 200 rpm (Model GFL 3005, Delitzsch, Germany). The extraction was carried out under light protected conditions (25). Then samples were filtered through Whatman No.1 filter paper and filtrate stored at−20°C until analysis.

TAC was determined using 2,2-diphenyl-1-picrylhydrazyl (DPPH) and ferric reducing antioxidant power (FRAP) methods. TAC was expressed as Trolox equivalent (TE)/g of sample. The DPPH method performed according to Ruiz-Torralba et al. (26). 250 μL of DPPH solution were added to 25 μL of extracted sample and deionized water were added to reach 10 mL final volume. The absorbance was measured at 515 nm. The FRAP method was conducted by the procedure described by Benzie and Strain (27). 25 μL of the acetone extract samples were mixed with 900 μL of freshly prepared FRAP solution and the final volume was adjusted to 10 mL with deionized water. Absorbance was measured at 593 nm.

#### 2.2.5 Water absorption index (WAI) and water solubility index (WSI)

WAI and WSI of untreated and treated grey pea flour samples were determined following procedures described by Du et al. (28). 2.5 g of flour were suspended in 30 mL distilled water in a pre-weighed centrifuge tube and cooked in a water bath for 30 min at 70°C. After cooling to room temperature, the mixture was centrifuged at 3000 × g for 20 min (Centrifuge 5804R, Eppendorf, Hamburg, Germany). The supernatant was transferred into pre-weighed aluminum containers to determine its solid content by evaporating the supernatant in an oven at 105°C overnight. The sediment was weighed. WAI and WSI were calculated using Equations 1, 2:


(1) $$WAI\;\left(g/g\right)=\frac{\mathrm{Weight of sediment}}{\mathrm{Weight of flour sample}}$$

(2) $$WSI\;\left(\frac{g}{100g}\right)=\frac{\mathrm{Weight of dissolved solids in supernatant}\times 100}{\mathrm{Weight of flour sample}}$$

#### 2.2.6 Color characteristics

Color measurements of flour samples were carried out using a portable spectrophotometer (Konica Minolta CM-700d/CM-600d, Tokyo, Japan). The recorded parameters were L*, a* and b*. The L*value indicates lightness ranging from 0 (dark) to 100 (light). The a* value represents green-red spectrum with positive number indicating redness and negative numbers indicating green color. The b* value represents the yellow-blue spectrum, with positive numbers indicating yellow color (29).

#### 2.2.7 Functional properties

##### 2.2.7.1 Water absorption capacity (WAC)

WAC was determined using the method described by Ferawati et al. (3). 3 g of sample were dispersed in 25 mL distilled water in a pre-weighed centrifuge tube and stirred every 5 min for 30 min and then centrifuged at 3000 × g for 25 min (Centrifuge 5804R, Eppendorf, Hamburg, Germany). After centrifugation, the supernatant was decanted and the excess moisture was removed by drying the samples in an oven (Termaks, TS4057, Bergen, Norway) at 50°C for 25 min. The tube was then reweighed, and the WAC was expressed as grams of water bound per gram of the sample on a dwb.

##### 2.2.7.2 Oil absorption capacity (OAC)

OAC was measured using the method described by Kaur and Singh (30). 0.5 g of sample were dispersed in 6 mL corn oil in a pre-weighed centrifuge tube and stirred for 1 min and left for 30 min before being centrifuged at 3000 × g for 25 min (Centrifuge 5804R, Eppendorf, Hamburg Germany). After centrifugation, the oil layer was removed, and the tube was inverted for 25 min to drain excess oil before being re-weighed. The OAC was expressed as grams of oil bound per gram of the sample on a dwb.

##### 2.2.7.3 Emulsion activity (EA) and emulsion stability (ES)

Emulsifying properties of flours were determined according to the method of Ferawati et al. (3). 3.5 g of flour sample were homogenized at 19000 rpm for 30 s in 50 mL distilled water. Then 25 mL peanut oil were added, and the mixture was homogenized again for 30 s. Another 25 mL of peanut oil were then added, and the mixture was homogenized for 90 s. The emulsion was evenly divided and transferred into two 50 mL centrifuge tubes and centrifuged at 1100 × g for 5 min. To determine emulsion stability the same method as described above was used to prepare the samples. The emulsified samples were heated for 15 min at 85°C in a water bath. Then cooled and centrifuged at 1100 × g for 5 min. EA and ES were calculated using Equation 3:


(3) $$\begin{array}{l} \mathrm{Emulsion activity}\;\\ \mathrm{and stability}\;\left(\mathrm{EA,ES,}\%\right)=\frac{\mathrm{Volume of emulsified layer}}{\mathrm{Total volume of emulsion}}\times 100 \end{array}$$

##### 2.2.7.4 Least gelation concentration (LGC)

The least gelation concentration was determined following the method described by Ferawati et al. (3). Suspension of grey pea flour samples at concentrations of 2, 4, 6, 8, 10, 12, 14, 16, 18, and 20% (w/v) were prepared and heated for 60 min in a boiling water bath. The test tubes were cooled immediately under cold running water and further cooled at 4°C for 2 h. The least gelation concentration is the concentration at which the samples did not fall or slip when the test tube was inverted.

#### 2.2.8 Pasting properties

The pasting properties of treated and untreated grey pea flours were studied by using a rapid visco analyzer (RVA, Perten 4,500, Stockholm, Sweden). A suspension of 3.5 g flour in 25 g of distilled water was prepared, adjusted to compensate for 14% moisture basis correction of the sample. The measurement protocol included 1 min of mixing, stirring, and warming up to 50°C at 160 rpm followed by 222 s of heating up to 91°C, 150 s of holding at 91°C, and then 228 s of cooling back down to 50°C, at the same rate as the heating. From the pasting curve, pasting temperature, peak viscosity, trough viscosity, breakdown, final viscosity, and setback were measured.

### 2.3 Statistical analysis

Processing trials were conducted in duplicate, further, untreated and treated samples were analyzed also in duplicate (*n* = 2), duplicate processing trials, duplicate analyses. Results are reported as mean ± standard deviation (SD). Paired *t*-tests were conducted to evaluate differences between whole and dehulled peas for each parameter under each treatment (i.e., TPC in fermented whole peas vs. TPC in fermented dehulled peas). One-way ANOVA followed by post-hoc Tukey analyses were used to determine significant differences between the reported parameters as a function of the type of treatment (i.e., comparison of TPC in raw, dehulled, germinated and fermented grey pea flour). Pearson correlations were computed to evaluate the associations between TAC obtained by two methods and the results of TPC in grey peas after each process. The level of significance was set at *p* < 0.05, statistical analyses were carried out using SPSS Statistics software version 26 (SPSS Inc., IBM Corporation, Armok, USA). Unpaired t-test was computed, to investigate differences on pH and lactic acid produced during fermentation, using GraphPad Prism 9 (GraphPad Software, Boston, MA, USA).

## 3 Results

### 3.1 Effect of fermentation on pH and acidity

**Figure 2:**
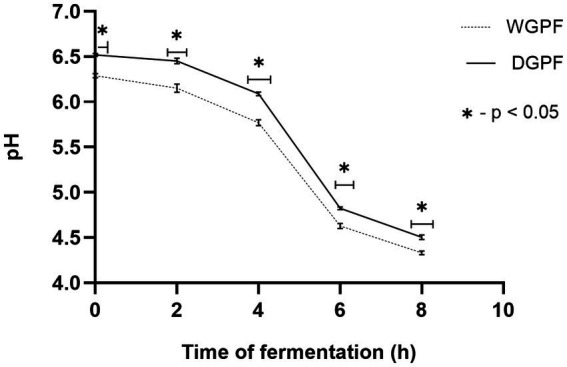
Changes in pH in whole and dehulled grey pea flour during fermentation. Significant differences are shown by * at *p* < 0.05 level. WGPF, whole grey pea flour; DGPF, dehulled grey pea flour.

**Figure 3:**
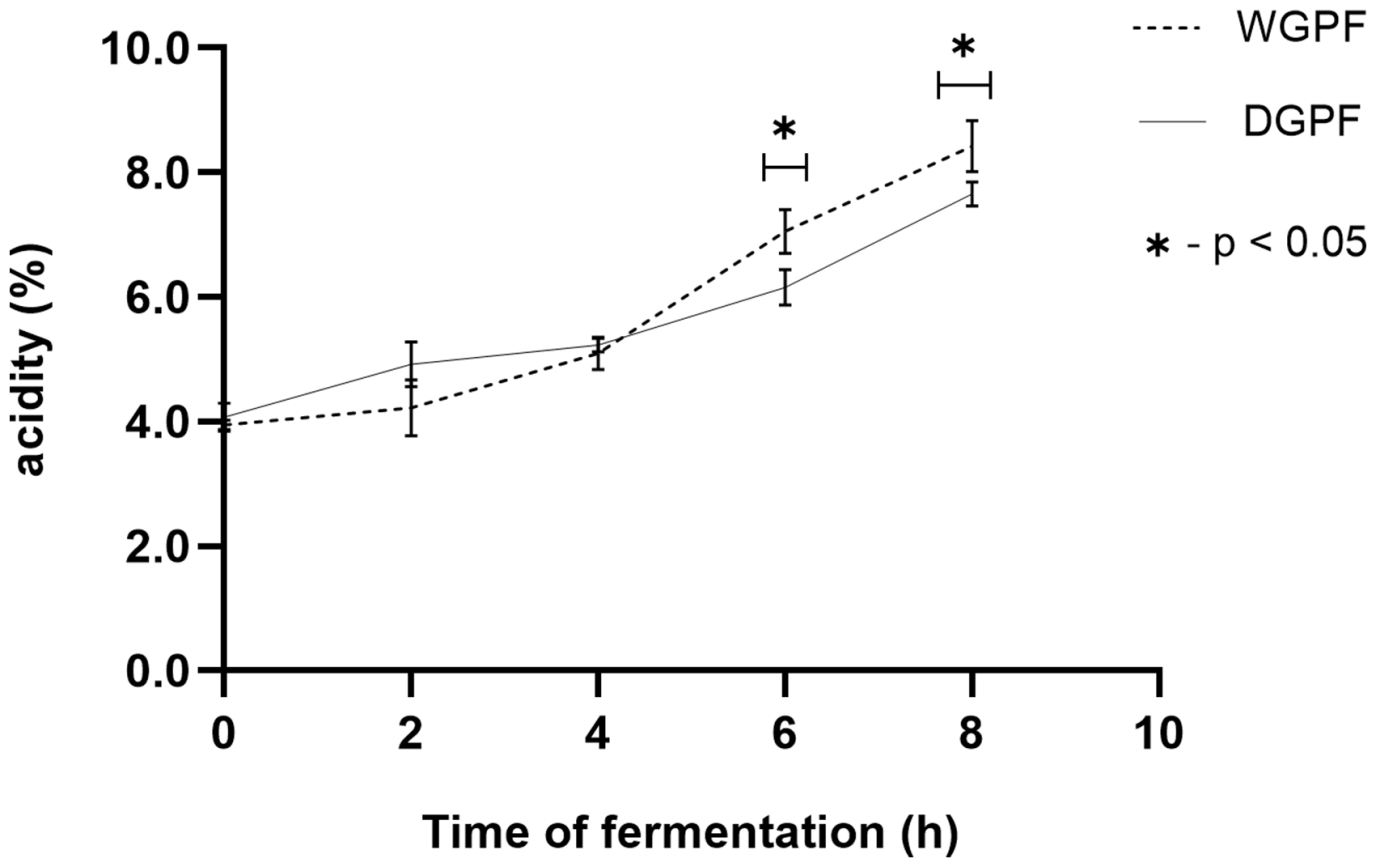
Changes in lactic acid content in whole and dehulled grey pea flour during fermentation. Significant differences are shown by * at *p* < 0.05 level. WGPF, whole grey pea flour; DGPF, dehulled grey pea flour; TTA, total titrable acidity.

The effect of fermentation on pH and total acidity of whole and dehulled grey pea flour is shown in Figures 2,3. Initial pH for whole and dehulled grey pea flour was recorded as 6.29 ± 0.02 and 6.52 ± 0.02, respectively. At the end of fermentation, pH dropped to 4.33 in whole flour, and to 4.5 in dehulled flour. Lactic acid content increased from 0.04 to 0.076% in whole flour and to 0.084% in dehulled flour. The results for pH showed significant differences between whole and dehulled flour at every 2 h intervals.

### 3.2 Processing effect on protein, TPC and TAC

The results for the moisture and protein content determination are shown in Table 1. Moisture content decreased in the fermented and 24 h germinated samples while the 48 h germinated sample retained nearly the same moisture level as raw flour. The protein content of flour ranged from 22.5 to 25.8%, with the raw whole flour having the lowest and the fermented dehulled flour having the highest protein content. The protein content changed significantly in dehulled treated flours compared to dehulled raw flour, after 24 h germination *p* = 0.003, after 48 h germination *p* = 0.002 and after fermentation *p* < 0.001.

**Table 1:** Moisture, protein content, total phenolic content and antioxidant capacity of raw, germinated and fermented grey pea flour, whole and dehulled.

	Raw		Germinated 24 h		Germinated 48 h		Fermented	
	Whole	Dehulled	Whole	Dehulled	Whole	Dehulled	Whole	Dehulled
Moisture (%)	10.6 ± 0.01^aC^	9.4 ± 0.1^bZ^	7.9 ± 0.55^aB^	7.0 ± 0.36^bY^	10.5 ± 0.52^aC^	10.0 ± 0.71^bAZ^	5.9 ± 0.25^aA^	5.8 ± 0.00^aX^
Protein (%)	22.5 ± 0.34^aA^	24.6 ± 0.29^bX^	23.7 ± 0.21^aBC^	25.7 ± 0.39^bY^	23.4 ± 0.52^aB^	25.8 ± 0.09^bY^	24.3 ± 0.15^aC^	25.8 ± 0.25^bY^
TPC (mgGAE/100 g)	142 ± 0.03^aC^	35 ± 0.07^bX^	74 ± 0.04^aA^	45 ± 0.18^aX^	116 ± 0.05^aB^	65 ± 0.22^bX^	227 ± 0.01^aD^	107 ± 0.10^bY^
TAC-FRAP (mgTE/100 g)	151.5 ± 6.4^aA^	53.6 ± 1.8^bX^	146.2 ± 4.2^aA^	56.3 ± 1.9^bXY^	180.9 ± 2.2^aB^	62.4 ± 3.7^bY^	204.1 ± 4.7^aC^	80.4 ± 3.8^bZ^
TAC-DPPH (mgTE/100 g)	194.9 ± 6.9^aA^	110.8 ± 7.4^bX^	203.2 ± 4.0^aA^	111.6 ± 5.7^bX^	253.4 ± 3.7^aB^	151.8 ± 3.9^bY^	398.3 ± 4.1^aC^	271.4 ± 9.4^bZ^

For each parameter (each row), superscripts lowercase letters (a, b) indicate differences due dehulling, for raw, germinated (24 or 48) and fermented flours. Superscripts uppercase letters indicate differences due to processing (i.e., germination or fermentation), note that letters ABC denote differences due processing for whole pea flour and letters XYZ denote differences due processing for dehulled pea flour. Results are presented as mean ± SD and significant differences were computed at *p* < 0.05. TPC, total phenolic content; TAC-FRAP, total antioxidant activity by FRAP; TAC-DPPH, total antioxidant activity by DPPH.

Total phenolic content of raw and treated grey pea flour is expressed as gallic acid equivalents (mg of GAE/100 g sample). The results are presented in Table 1. Significant differences were found in the TPC after treatment methods. Fermented whole flour showed the highest TPC with 227 mg GAE/100 g sample, 1.6-fold higher than raw whole pea flour while raw dehulled grey pea flour had the lowest TPC at 35 mg GAE/100 g sample. Dehulling led to a significant reduction (*p* < 0.001) in the TPC of raw flours, which resulted in a 4-fold decrease of TPC in dehulled pea flour.

The TAC results using two different methods, FRAP and DPPH, are presented in Table 1. In both methods fermented whole peas had the highest TAC value with 204 ± 4.7 and 398 ± 4.1 mgTE/100 g in FRAP and DPPH, respectively, while the lowest value was observed in raw dehulled peas with 53.6 ± 1.8 mgTE/100 g in FRAP method and 110.8 ± 7.4 mgTE/100 g in DPPH method. Dehulling significantly decreased TAC in both methods. Regarding germination, a significant increase was observed only in 48 h germinated flours in both flours made of whole and dehulled peas. Additionally, a significant correlation was found between TPC and TAC using both methods with *r* = 0.810 for TPC vs. TAC-FRAP and *r* = 0.898 for TPC vs. TAC-DPPH.

### 3.3 Water absorption index (WAI) and water solubility index (WSI)

Table 2 presents the results for the WAI and WSI of raw and treated grey pea flours. The WAI ranged from 2.89 to 3.33 (g/g), with raw dehulled flour having the highest value. No significant difference was observed in WAI between raw whole flour and other treatments as well as between raw dehulled flour and other dehulled treatments. Raw dehulled grey pea flour had the highest WSI with 24.50 (g/100 g). However, this value was not significantly higher than those obtained for 24 and 48 h dehulled germinated grey pea flours.

**Table 2:** Water absorption index (WAI), water solubility index (WSI) and color characteristic of raw, germinated and fermented grey pea flour, whole and dehulled.

		Raw		Germinated 24 h		Germinated 48 h		Fermented	
		Whole	Dehulled	Whole	Dehulled	Whole	Dehulled	Whole	Dehulled
WAI (g/g)		2.89 ± 0.37^aA^	3.33 ± 0.12^bX^	3.30 ± 0.07^aA^	3.07 ± 0.05^bX^	3.13 ± 0.03^aA^	3.15 ± 0.20^aX^	3.29 ± 0.10^aA^	3.13 ± 0.15^bX^
WSI (g/ 100 g)		19.90 ± 0.38^aB^	24.50 ± 0.50^bY^	20.00 ± 0.73^aB^	23.90 ± 0.50^bY^	18.80 ± 0.73^aB^	23.70 ± 0.38^bY^	8.40 ± 0.56^aA^	11.00 ± 0.23^bX^
Hunter color values	L*	82.20 ± 0.57^aBC^	88.37 ± 1.04^bYZ^	83.83 ± 0.62^aC^	89.20 ± 0.59^bZ^	81.89 ± 1.18^aB^	87.10 ± 0.17^bY^	70.98 ± 0.56^aA^	84.31 ± 0.21^bX^
	a*	1.52 ± 0.07^aB^	1.21 ± 0.06^bY^	0.82 ± 0.14^aA^	0.46 ± 0.02^bX^	1.08 ± 0.21^aA^	0.65 ± 0.16^bX^	3.67 ± 0.16^aC^	1.88 ± 0.08^bZ^
	b*	14.91 ± 1.22^aB^	18.15 ± 0.26^bY^	12.50 ± 0.66^aA^	15.42 ± 0.22^bV^	12.05 ± 0.44^aA^	16.69 ± 0.04^bX^	11.45 ± 0.42^aA^	20.19 ± 0.25^bZ^

For each parameter (each row), superscripts lowercase letters (a, b) indicate differences due dehulling, for raw, germinated (24 or 48) and fermented flours. Superscripts uppercase letters indicate differences due to processing (i.e., germination or fermentation), note that letters ABC denote differences due processing for whole pea flour and letters XYZ denote differences due processing for dehulled pea flour. Results are presented as mean ± SD and significant differences were computed at *p* < 0.05.

### 3.4 Color characteristics

Hunter color values (L*, a*, b*) of flour samples are shown in Table 2. Dehulling affected the hunter color values in all flour samples compared to their whole flour. Fermented whole flour showed the lowest L* and b* value, while having the highest a* value.

### 3.5 Functional properties

It can be seen in Table 3, that most of the functional properties of grey pea flour had changed by different processing methods. The WAC of different flours ranged from 0.84 to 1.54 gwater/g DM and the fermented whole grey pea flour showed a significantly higher WAC than other flours. The OAC of raw and treated flours ranged from 0.81 to 1.06 g oil/g DM, with the 48 h germinated whole flours having the highest and the dehulled fermented flours having the lowest capacity to absorb oil. Germination did not significantly affect EA and ES. Fermentation resulted in a poor EA (3.49 ± 0.47% and 3.25 ± 0.24% in whole and dehulled flour respectively) and ES (4.62 ± 0.49% in whole and 4.36 ± 0.57% in dehulled flour), while significantly improved the WAC (1.54 ± 0.01 and 1.24 ± 0.04 g water/g DM in whole and dehulled flour respectively). Highest ES (57.64 ± 2.36%) was found in 48 h whole germinated flour. When looking at the effect of dehulling, significant improvements were not found compared to the whole flour in treated and untreated dehulled grey pea flours. The LGC values ranged from 10 to 12%. The LGC for both whole and dehulled flour remained consistent across different treatments indicating that dehulling, germination or fermentation did not impact the gelling capacity of grey pea flour.

**Table 3:** Functional properties of raw, germinated and fermented grey pea flour, whole and dehulled, results are presented in means ± SD in dry matter (DM).

	Raw		Germinated 24 h		Germinated 48 h		Fermented	
	Whole	Dehulled	Whole	Dehulled	Whole	Dehulled	Whole	Dehulled
WAC (g water/ g)	1.13 ± 0.02^aC^	0.84 ± 0.05^bX^	0.95 ± 0.01^aA^	0.84 ± 0.01^bX^	1.09 ± 0.02^aB^	0.97 ± 0.01^bY^	1.54 ± 0.01^aD^	1.24 ± 0.04^bZ^
OAC (g oil/ g)	0.94 ± 0.03^aB^	0.90 ± 0.04^aXY^	0.98 ± 0.07^aBC^	0.96 ± 0.02^aY^	1.04 ± 0.02^aC^	1.06 ± 0.07^aZ^	0.83 ± 0.01^aA^	0.81 ± 0.03^aX^
EA (%)	49.28 ± 0.92^aB^	49.26 ± 0.47^aY^	48.30 ± 0.48^aB^	48.65 ± 0.24^aY^	48.04 ± 0.38^aB^	48.65 ± 0.24^aY^	3.49 ± 0.47^aA^	3.25 ± 0.24^aX^
ES (%)	53.79 ± 1.29^aBC^	54.10 ± 2.00^aY^	51.70 ± 3.44^aB^	52.79 ± 2.96^aY^	57.64 ± 2.36^aC^	54.44 ± 5.03^aY^	4.62 ± 0.49^aA^	4.36 ± 0.57^aX^
LGC (%)	12%^a^	12%^a^	10%^a^	10%^a^	10%^a^	10%^a^	12%^a^	12%^a^

For each parameter (each row), superscripts lowercase letters (a, b) indicate differences due dehulling, for raw, germinated (24 or 48) and fermented flours. Superscripts uppercase letters indicate differences due to processing (i.e., germination or fermentation), note that letters ABC denote differences due processing for whole pea flour and letters XYZ denote differences due processing for dehulled pea flour. Results are presented as mean ± SD and significant differences were computed at *p* < 0.05.WAC, water absorption capacity, OAC, oil absorption capacity, EA, emulsion activity, ES, emulsion stability, LGC, least gelation concentration.

### 3.6 Pasting properties

The results of the rapid visco analyzer for the raw and treated flours are presented in Table 4. The pasting temperature of flours was not significantly affected by the type of processing method except for the fermented dehulled flour sample, in which higher pasting temperature was observed (86.93 ± 0.03°C). Peak viscosities of flour were affected by both dehulling and processing methods. Dehulling increased peak viscosity in raw and treated samples. The highest peak viscosity was observed in 48 h germinated samples (1,061 ± 2.12 cP in whole and 1,308 ± 2.12 cP in dehulled flour) while fermentation resulted in lowest peak viscosity (291 ± 2.12 and 633 ± 1.41 cP in whole and dehulled flour respectively). The lowest breakdown value was found for 24 h germinated dehulled flour (5.50 ± 0.71 cP). In contrast the highest breakdown values were observed for 48 h germinated and fermented dehulled flours with 80 ± 0.00 and 82 ± 2.82 cP, respectively. In general, higher viscosities were obtained with different treatment methods except for the fermentation process, which on contrary caused a decrease in viscosities and resulted in the least paste stability. Additionally, 48 h germination had greater impact on viscosities compared to 24 h germination.

**Table 4:** Pasting properties of raw, germinated and fermented grey pea flour, whole and dehulled.

	Raw		Germ 24 h		Germ 48 h		Fermented	
	Whole	Dehulled	Whole	Dehulled	Whole	Dehulled	Whole	Dehulled
Pasting temperature (°C)	76.93 ± 0.53^aA^	76.87 ± 0.53^aX^	77.58 ± 0.53^aA^	76.85 ± 0.49^bX^	76.55 ± 0.07^aA^	76.15 ± 0.63^aX^	76.83 ± 0.53^aA^	86.93 ± 0.03^bY^
Peak viscosity (cP)	871 ± 13.43^aB^	942 ± 8.48^bY^	878 ± 1.42^aB^	934 ± 4.24^bY^	1,061 ± 2.12^aC^	1,308 ± 2.12^aZ^	291 ± 2.12^aA^	633 ± 1.41^bX^
Trough viscosity (cP)	848 ± 15.55^aB^	930 ± 4.95^aY^	863 ± 1.41^aB^	928 ± 4.95^bY^	1,025 ± 1.41^aC^	1,228 ± 2.12^aZ^	241 ± 0.70^aA^	551 ± 4.24^bX^
Breakdown (cP)	23.5 ± 2.12^aAB^	11.5 ± 3.53^aX^	15 ± 0.00^aA^	5.50 ± 0.71^bX^	36 ± 0.70^aBC^	80 ± 0.00^aY^	50 ± 1.41^aC^	82 ± 2.82^aY^
Final viscosity (cP)	1,369 ± 2.82^aB^	1,434 ± 9.89^bY^	1,312 ± 2.12^aB^	1,414 ± 13.44^aY^	1,415 ± 4.94^aB^	1,677 ± 3.53^aZ^	463 ± 4.94^aA^	893 ± 2.12^bX^
Setback (cP)	521 ± 12.72^aC^	503 ± 4.94^aZ^	449 ± 3.53^aB^	486 ± 8.49^aZ^	389 ± 3.53^aB^	449 ± 1.41^aY^	222 ± 4.24^aA^	342 ± 6.36^bX^

For each parameter (each row), superscripts lowercase letters (a, b) indicate differences due dehulling, for raw, germinated (24 or 48) and fermented flours. Superscripts uppercase letters indicate differences due to processing (i.e., germination or fermentation), note that letters ABC denote differences due processing for whole pea flour and letters XYZ denote differences due processing for dehulled pea flour. Results are presented as mean ± SD and significant differences were computed at *p* < 0.05.

## 4 Discussion

### 4.1 Effect of fermentation on pH and total acidity

Fermentation resulted in a decrease in the pH and increase in lactic acid content for both whole and dehulled flour. These changes can be attributed to microbial activity, particularly the dominance of lactic acid bacteria, which degrade carbohydrates and acidify the products. It is reported that lactic acid produced during fermentation with lactic bacteria acidifies the products, inhibiting spoilage bacteria and thus ensuring its preservation, a key factor in its popularity within the food industry (31).

Interestingly, alkaline fermentation of legumes has been previously reported, with the increase in pH attributed to protein degradation by *Bacillus* spp. During this process, sources of carbon and nitrogen are used by the bacteria to produce ammonium hydroxide and ammonia, resulting in high pH values and the characteristic odor of alkaline fermented foods (i.e., natto, douchi), which are mainly produced from soybeans (32). In contrast, in this study, the use of lactic acid bacteria for the fermentation of grey pea resulted in a decrease in pH due to increased acidity from lactic acid production. The selection of raw materials, starter cultures and fermentation conditions are key parameters influencing the course of fermentation and the characteristics of the final fermented product. Additionally, lactic bacteria may enhance sensory characteristics of fermented foods by generating desirable aroma components and reducing off-flavors, contributing to the overall improvement in taste and quality (33). The highest pH reduction occurred between 4 and 6 h fermentation process, this is likely the time needed to adapt fermentation condition by endogenous microbes (34). Fermentation of both raw and dehulled grey pea flour showed similar trends in acidity during fermentation with no significant difference between the two flours at the end of fermentation. Therefore, dehulling does not appear to affect fermentation outcomes in terms of acidity.

### 4.2 Moisture and protein content

Higher moisture content in 48 h germinated sample compared to 24 h germination may be due to increased water uptake by grey pea seeds in order to carry out the metabolic processes during germination, which resulted in more hydrated cells within the seeds, similar trend was shown for germination of chickpeas flour (35). It is important to monitor the moisture content in flours, as higher moisture levels can impact the food product’s characteristics including physical appearance, texture, taste, weight. Additionally, moisture content affects factors such as shelf-life, freshness, quality and resistance to bacterial contamination.

Dehulling significantly increased protein content of raw and processed grey pea flour. This is because seed coat (hull) of pulses contains little to no protein. Removing the hull increases the concentration of the endosperm, thereby proportionally increasing the protein content in the dehulled seed (36, 37). The findings of this research are in agreement with the results obtained by Wang et al. (21) in various lentils varieties and by Pal et al. (36) in horsegram pulses. The results of this study showed a 5.3% increase in protein content in whole pea flour during 24 h germination and 4% after 48 h germination. It has been suggested that during germination, hydrolytic activities of the enzymes increased due to breakdown of proteins. The relative increase of protein in fermented grey peas can be attributed to the natural increment in bacterium biomass and the conversion of the inorganic nitrogen to organic nitrogen. This effect combined with the reduction of carbohydrates in the form of sugars consumed by bacteria during fermentation, may contribute to the higher protein content in the fermented products (38). Similar increase in the protein content (4.47%) of germinated grass pea flour was reported by Lakshmipathy et al. (39). Other authors have also reported an increase in protein during fermentation of pigeon pea 3.67% after 1 day and 9.63% after 5 days. These pigeon peas were boiled and dehulled and underwent natural fermentation (40).

### 4.3 Total phenolic content (TPC)

Dehulling significantly decreased the TPC in raw flour and this decreasing trend was observed in all treatments (ranging from 40.84–75.35%). Singh et al. (41) noted that the seed coat (hull) of pulses which acts as a protective layer for the cotyledons contains high concentrations of phenolic compounds. Consequently, dehulling of pulses removes substantial amounts of polyphenols. The reduction in TPC observed in germinated whole flour could be due to the increased activity of polyphenol oxidase and other catabolic enzymes. Additionally, the activation of enzymes during germination leads to hydrolysis of various components including phenolic compounds (42). Guajardo-Flores et al. (43) reported a 58.33% decrease in raw and 10% increase in 5-day germinated dehulled black bean flour. Similarly, Lakshmipathy et al. (39) observed a 14.4% reduction in TPC for dehulled and a 15.34% increase in 48 h germinated grass pea flour. The TPC reduction in raw dehulled grey pea flour in this study was higher (75.35%) than the one in the mentioned studies. During germination of whole grey pea flour TPC decreased by 47.98 and 18.3% for 24 and 48 h, respectively, while in dehulled grey pea flour TPC increased by 28.75 and 85.71% for 24 and 48 h germination, respectively. Similarly, Navyashree et al. (44) reported TPC reduction (47.72%) in 48 h germinated white finger millet. As germination time increased the TPC was also increased (from 74 ± 0.04 to 116 ± 0.05 mgGAE/100 g in whole and from 45 ± 0.18 to 65.022 mgGAE/100 g in dehulled flour). Similar results were obtained for chickpea flour from 130.41 ± 2.67 to 245.25 ± 2.61 mgGAE/100 g with germination time from 12 to 48 h (35). A significant increase in TPC in fermented samples (59.86% in whole and 205% in dehulled) might be due to the degradation of polymeric phenolic compounds by proteolytic enzymes into simpler, more biologically active compounds which are then released as soluble phenolic compounds. Additionally, fermentation can loosen the lignocellulosic matrix, resulting in the release of phenolic compounds from an inaccessible state (11, 45). Çabuk et al. (11) reported 88% increase in TPC in pea protein concentrate after 9 h fermentation with *L. plantarum.* Additionally, increases of 84.9 and 90.6% in TPC were observed after 48 h of fermentation with *L. plantarum* and natural fermentation of soybean flour, respectively, as reported by Fernandez-Orozco et al. (46).

### 4.4 Total antioxidant capacity (TAC)

The antioxidant capacity was measured through two different methods, the FRAP assay which assesses metal reducing ability in the presence of antioxidant and DPPH assay which is based on antioxidant ability to scavenge free radical (47). Dehulling significantly decreased antioxidant activity in raw flour by 64.62% (FRAP) and 43.15% (DPPH) and this decreasing trend was observed across all treatments likely due to hull removal. Phenolic compounds which are concentrated in seed coat are closely correlated to antioxidant capacity. Consequently, removing the hull which contains high amount of these phenolic compounds results in a decrease in antioxidant capacity (43). The results are aligned with findings of Nelom et al. (48) where TAC reduction varied from 55.72 to 67.76% for dehulled cowpea when TAC was measured through DPPH method. Lower TAC reduction (13.08%) was reported by Lakshmipathy et al. (39) for grass pea flour using DPPH assay. The effect of germination on increasing antioxidant capacity could be explained by synergistic effect with phenolic compounds. Additionally enzymatic reaction during germination can enhance TAC due to formation of phenolic compounds from seed coats and cotyledons (49). In this study TAC in 48 h germinated whole flour increased by 19.4 and 30% using FRAP and DPPH methods, respectively. TAC increase (16.45%-DPPH) due to germination was reported by Lakshmipathy et al. (39) when grass pea was germinated for 48 h. Mao et al. (50) also reported 8.87 to 24.79% increase for different 72 h germinated chickpeas varieties using FRAP and 7.66 to 168% increase using DPPH assays. In this study, antioxidant activity improved after fermentation by 34.72 and 50% using FRAP, 104.36 and 145.4% using DPPH of whole and dehulled grey pea flour, respectively. It has been argued that microbial hydrolysis occurring during fermentation increases phenolic compounds and flavonoids. This hydrolysis may cause that bound phenolics are converted into free forms, resulting in a higher antioxidant level (51). In addition, Fermentation causes the structural disintegration of plant cell walls, resulting in the release of diverse antioxidant compounds (52). Okechukwu (53) reported that the DPPH antioxidant activity of ethanolic extract of naturally fermented pigeon pea at room temperature for 7-days increased from 0.810 to 1.014 mg/mL. The results in Table 1 indicate a correlation between TPC and TAC. However, this relation is complex and involves various factors. For instance, other substances can contribute to antioxidant properties beyond phenolic compounds. Moreover, Different analytical methods for assessing antioxidant capacity can lead to varying results.

### 4.5 Water absorption index (WAI) and water solubility index (WSI)

There was an increase (14.18 and 8.3%), however not significant, in WAI in 24 and 48 h germinated samples, respectively, compared to whole raw flour, which may be due to more hydrophilic sites exposed to water after germination. According to Du et al. (28) WAI is related to the hydrophilicity and gelation capacity of starch and protein as biomacromolecules in flour. Similar increase in WAI in 24 h germinated faba bean flour (2.79 to 3.13) was reported by Kumar et al. (54). Similar increase was found in grey pea whole flour after fermentation (2.89 to 3.29). Onweluzo and Nwabugwu (55) suggested that during fermentation process high molecular weight proteins and carbohydrates are hydrolyzed into smaller and more soluble components, thereby increasing WSI. However, a significant decrease (57.79 and 55.1%) in the WSI was observed in fermented grey pea flour. These findings are in agreement with results obtained by Toor et al. (56) who reported a decrease in WSI in fermented chickpea (3%) and pigeon pea (9%) flours. This reduction in WSI could be attributed to utilization of soluble compounds by microorganisms for their growth as discussed by Ilowefah et al. (17). The higher protein content might also explain the elevated WSI observed in dehulled fermented flour. There was also observed a significant increase in WSI in all dehulled samples, since WSI, is related to the presence of soluble molecules, dehulling step might have resulted in the increased solubility of grey pea flour by eliminating the effect of the seed coat on solubility, which is mainly composed of insoluble fiber.

### 4.6 Color characteristics

Color is a crucial characteristic for consumer appeal, particularly when incorporating as an ingredient in the final product (57). The higher L* value in dehulled flours ranging from 84.31 to 89.20 indicated a visually lighter color. This could be attributed to the reduction of phenolic and chlorophyl due to seed coat removal (58). This is in line with the TPC results that were significantly lower in dehulled samples. The observed increase, although not significant, in the lightness (L* value) of germinated flour may be attributed to the dissociation of colored pigments during the soaking process (44). It was also indicated that during germination of whole flour, non-enzymatic browning occurs due to the transfer of color pigments from seed coat to endosperm (35). Thus, the lightness of germinated flour can fluctuate based on the stages of soaking and germination. The reduction in a* and b* values in germinated flour could be attributed to changes in carbohydrates and protein hydrolysate (57). Similarly Lakshmipathy et al. (39) reported a reduction in a* and b* values in germinated grass pea flour. In fermentation the color variations could be attributed to the degradation of pigments (56). Since all treatment methods involve a heating step, heat treatment can impact each of the color values to some extent.

### 4.7 Functional properties

#### 4.7.1 Water absorption capacity (WAC) and oil absorption capacity (OAC)

WAC of the flours is associated with the presence of hydrophilic components (30). Different protein conformations and hydrophilic carbohydrates fractions in flours contribute to variations in WAC (59). In this study, there was a significant reduction in WAC in both raw (25.66%) and treated dehulled flour which could be attributed to the removal of seed coat. The fibers present in hull bind and hold water and their absence leads to a decreased ability of the flour to retain water (60). Similar results were reported for dehulled grass pea flour (11.16%) by Lakshmipathy et al. (39). Lower WAC in germinated flour compared to whole raw flour (15.93 and 3.54% in 24 and 48 h germination respectively) may result from the reduction of hydrophilic points due to enzymatic degradation of starch and fiber as seen in germinated black chickpea flour by Kumar et al. (57). Conversely Ferawati et al. (3) reported an increase in WAC in 24 and 48 h germinated grey pea. An increase in WAC in 48 h germinated dehulled flour which was attributed to the enhancement of water binding sites resulting from macromolecules modification (61). Fermentation caused an increase in WAC (36.28 and 47.62% in whole and dehulled flour respectively), in previous studies it was observed an increase in WAC in fermented chickpeas and red beans suggesting that microbial protease enzymes breakdown peptide bonds during fermentation, leading to an increase in hydrophilic groups in proteins and low molecular weight proteins (62, 63).The OAC is influenced by the binding of lipids to the hydrophobic amino acid side chains and their availability on the protein surface. The observed improvement (10.63 and 17.77%) in the OAC of 48 h germinated whole and dehulled flour, respectively, could be due to the changes in the conformation of the protein molecules which may have resulted in more exposure of the non-polar residues from the interior to the surface and increase in the surface availability of these hydrophobic amino acids (60). The decrease or increase of OAC during fermentation depends on the surface availability of hydrophobic amino acids as fat droplets bind with non-polar molecules. Therefore, any changes in protein molecular structure can result in an increase or decrease on OAC (56).

#### 4.7.2 Emulsifying properties

Emulsifying properties of pulse flours are generally assessed by two parameters, emulsifying activity (EA) and emulsifying stability (ES). Germination is thought to enhance EA and ES through the dissociation and partial unfolding of polypeptides, exposing hydrophobic amino acid sites. This exposure enhances the hydrophobic interactions between peptide chains and lipid droplets, significantly boosting the availability of protein volume and surface area (39). However, in this study germination did not improve emulsifying properties of grey pea flour. This observation aligns with findings by Ferawati et al. (3) where a similar decrease was obtained in germinated grey pea. This reduction could be attributed to changes in protein concentrations or alteration of hydrophobicity/hydrophilicity ratio and structural constraints of the proteins. These factors could affect protein’s ability to unfold and form a film around dispersed oil droplets (64).

Fermented flour showed a significant decrease in EA by approximately 93% and ES by around 92%. This reduction is attributed to the increase in hydrophobicity which affects protein’s ability to migrate to the oil–water interface. This migration is crucial for lowering interfacial tension and facilitating emulsion formation and unhydrolyzed proteins (65). In addition, during fermentation the concentration of water-soluble protein decreases affecting the emulsifying properties of fermented flour. EA of pea flour decreased to 3.39 m^2^/g after fermentation with *L. rhamnosus* L08 (66).

#### 4.7.3 Least gelation concentration (LGC)

Although, dehulling process contributed to higher protein concentration, potentially improving the formation of the three-dimensional network, no significant difference in LGC of whole and dehulled flours was obtained in this study. This could be attributed to the fact that the increase in protein concentration facilitates gelation due to more intermolecular interaction during heating. Alternatively, the complex carbohydrate present in the seed coat may have interfered with the formation of a continuous network of molecules suggesting that their impact on the final gelation was not substantial enough to significantly affect the LGC (67, 68).

### 4.8 Pasting properties

Pasting properties are crucial for various applications in the food industry, as they are influenced by the presence of starch, protein, amylase activity and amylose/amylopectin ratio in the flour (35, 69). The pasting temperature did not change significantly in germinated flour. The increase in viscosities of dehulled flour samples might be due to a higher proportion of starch compared to fibrous whole flours (70). Similar result was observed in dehulled chickpea and faba bean flour by Teferra et al. (70). For 48 h germinated samples the increase in viscosity can be related to changes in the ratio of amylose /amylopectin due to starch degradation during germination. Increased interaction of starch granules with amylolytic enzymes can result in lower amylose content, which happens due to the breaking of intact cell walls during germination (71). The increase in peak viscosity for germinated flour was attributed to starch granules swelling resulting from protein and fiber matrix loosening (61). Breakdown viscosity decreased in 24 h germinated flour (36.17 and 52.17% in whole and dehulled flour respectively), while it increased in 48 h germinated flour. Lower breakdown viscosity indicates good paste stability and strong shearing resistance. Final viscosity improved significantly in 48 h germinated dehulled flour. Setback viscosity decreased for both 24 and 48 h germinated flour indicating a high retrogradation tendency. These results are similar to findings for 48 h germinated bambara groundnut flours where peak viscosity, trough viscosity, breakdown viscosity, final viscosity and setback viscosity increased by 2, 1.46, 54.28, 3.99 and 19.24%, respectively (61). The decrease in breakdown, setback and final viscosity in 24 h germinated flour could be attributed to the degradation of starch granules and hydrolysis of amylopectin and amylose by enzymes during germination, which can lead to less entanglement between the chains (72).

In the fermented flour pasting viscosities decreased by 66.55, 71.58, 66.14 and 57.39% for peak, trough, final and setback viscosity, respectively, compared to raw flour with the exception of breakdown value which increased by 112% in whole flour. Li et al. (73) investigated the effect of yellow pea flour fermentation with five lactic acid bacteria strains on pasting properties. The pasting properties of fermented yellow pea flour were significantly lower compared to raw flour. For instance, yellow pea fermented with *Lactobacillus acidophilus* ATCC 43121 for 18 h showed 6.09, 1.91, 29.05, 6.63% and 22.42% reduction in peak, trough, breakdown, final and seatback viscosity, respectively. Having compared and discussed the pasting properties of grey pea flour samples, it is worth mentioning that pasting properties of flours are not only affected by starch but also non-starch components such as proteins, fat, and fiber and their interactions with starch can influence the performance of flours during pasting (23). Lower pasting properties obtained through fermentation may be favorable for certain applications in the food industry, where lower tendencies to retrograde are favorable, for examples in the formulation of soups and sauces, since they can experience loss of viscosity and precipitation due to retrogradation (30). A better understanding of interactions at the molecular level is required to better understand these results.

## 5 Conclusion

In conclusion, various processing methods—such as dehulling, germination, and fermentation—played crucial roles in modifying the bioactive and functional properties of grey pea flour. Germination and in particular, lactic fermentation significantly enhanced bioactive properties as measured by TPC and TAC. The main findings of this research indicate that fermentation of grey peas notably increased TPC and improved total antioxidant capacity as measured by both the FRAP and DPPH methods. Additionally, protein content showed increase following germination and fermentation of whole and dehulled grey pea flour. The impact of processing on functional properties varied; in some cases, such as germination, functional properties like pasting improved, while in others, such as fermentation, they were reduced. Depending on the intended purpose and desired properties of the final product, where grey peas are considered a major component, the obtained results provide a solid foundation for selecting a suitable processing method for grey peas and their successful incorporation into food formulations. Furthermore, the results could have policy implications by encouraging industries and farmer to increase grey pea production and processing.

## Data Availability

The raw data supporting the conclusions of this article will be made available by the authors, without undue reservation.
